# The Successful Rapid Adjustment of Blood Glucose in a Patient With Acute Coronary Syndrome, Renal Insufficiency, and Diabetes: A Case Report of Management Coordinated by Clinical Pharmacists and Clinicians

**DOI:** 10.3389/fphar.2020.00756

**Published:** 2020-05-21

**Authors:** Fang-Hong Shi, Long Shen, Mang-Mang Pan, Lin-Lin Ma, Chi Zhang, Zhi-Chun Gu, Jing Ma, Hao Li

**Affiliations:** ^1^Department of Pharmacy, Renji Hospital, School of Medicine, Shanghai Jiao Tong University, Shanghai, China; ^2^Department of Cardiology, Renji Hospital, School of Medicine, Shanghai Jiao Tong University, Shanghai, China; ^3^Institute for Drug Discovery, Griffith University, Brisbanei, Australia; ^4^Department of Endocrinology and Metabolism, Renji Hospital, School of Medicine, Shanghai Jiao Tong University, Shanghai, China; ^5^Department of Pharmacy, Clinical Research Center, Shanghai Children's Medical Center, Shanghai Jiao Tong University School of Medicine, Shanghai, China

**Keywords:** blood glucose management, acute coronary syndromes, renal insufficiency, diabetes, clinical pharmacists, coordination

## Abstract

Diabetes is a major cause of cardiovascular mortality in most countries. Intensive management of blood glucose is pivotal for alleviating disease progress and minimizing cardiovascular complications. In this study, we report a case of successful control of high blood glucose in a diabetes patient with acute coronary syndromes (ACS), hypertension, and renal insufficiency. This patient had five years of diabetes history and was hospitalized through an ACS emergency. Coronary angiography showed an acute anterior myocardial infarction (Killip Level I). The patient had extremely high blood glucose that ranged from 19.4 to 28.2 mmol/L on the first day in the hospital and experienced significant blood glucose fluctuations in the following three days. After two rounds of clinical pharmacist consultation, the patient's fasting blood glucose (FBG) target was achieved on the seventh day of his hospitalization and was well controlled afterward. The patient's postprandial blood glucose (PBG) target was achieved on the ninth day of hospitalization, and he was discharged when his blood glucose was well controlled and cardiac function had been fully assessed. Hence, we summarize a protocol that could be used to quickly adjust high blood glucose in hospitalized patients and report a new blood glucose management model coordinated by clinical pharmacists and clinicians.

## Background

In the past forty years, the prevalence of diabetes has increased dramatically, with significantly higher rates in urban areas such as Shanghai (Jia et al., 2007; Chan et al., 2009; Zimmet et al., 2014; Huang, 2016; Zimmet et al., 2016; Association, 2019a). More than 20% of people over 65 years suffer from diabetes worldwide, and more than 90% of patients with diabetes are type 2 diabetes (T2D) (Huang, 2016; Chatterjee et al., 2017). T2D is associated with an increased risk of coronary heart disease (Sarwar et al., 2010) and is the sixth leading cause of disability (Chatterjee et al., 2017; Collaborators., 2016). Intensive management of glucose is critical to slow down the disease progression and minimize the risk of cardiovascular complications (Holman et al., 2008; Holman et al., 2014; Gregg et al., 2016). Insulin therapy is an effective and less expensive choice for T2D (Lipska et al., 2017). T2D patients with fasting blood glucose (FBG) level higher than 13.9 mmol/L (250 mg/dl), hemoglobin A1c (HbA1c) level higher than 10%, and/or with severe symptoms of hyperglycemia are recommended with insulin therapy (Wallia and Molitch, 2014). We report one such case demonstrating a rapid and successful blood glucose management strategy coordinated by clinical pharmacists and clinicians on a 75-years-old patient who suffered from acute coronary syndrome (ACS), renal insufficiency, and diabetes.

## Case Presentation

**Figure 1:**
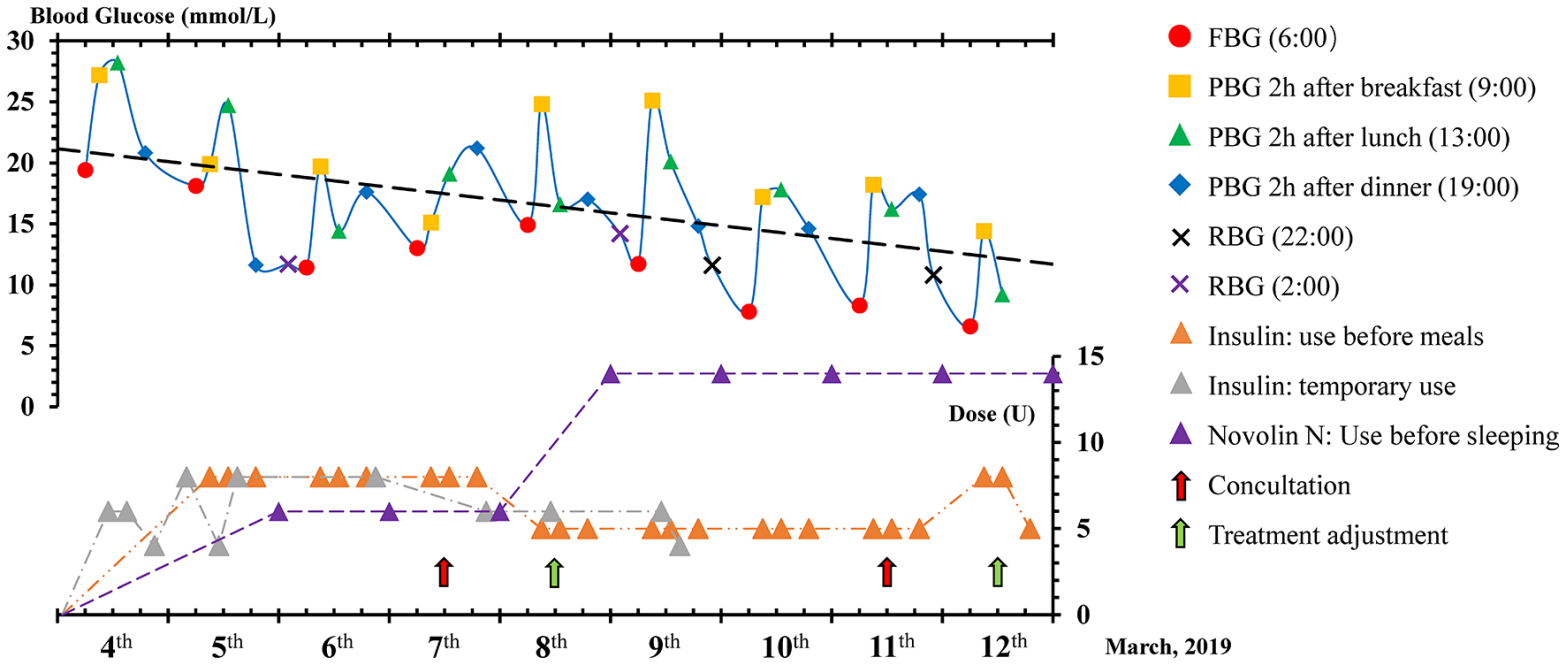
Dynamic changes in blood glucose and insulin dosage. FPG, Fasting Blood Glucose; PBG, Postprandial Blood Glucose; RBG, Random Blood Glucose.

**Figure 2:**
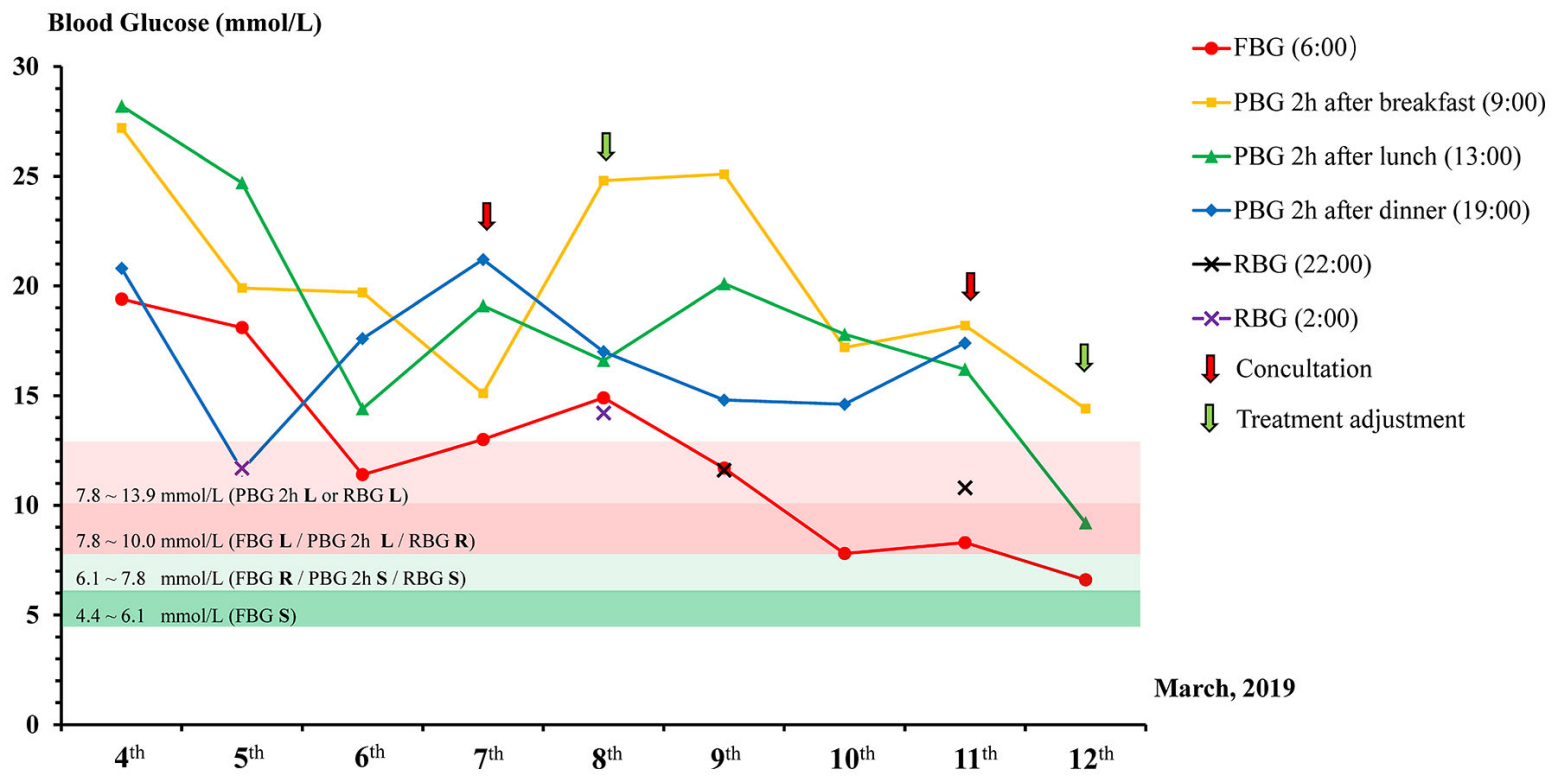
Dynamic changes of different blood glucose observation indicators. The target blood glucose range is labeled with a different color strip. FBG, Fasting Blood Glucose; PBG, Postprandial Blood Glucose; RBG, Random Blood Glucose; L, Less stringent; R, Reasonable; S, Stringent.

The patient in this study was a 75-year-old Chinese male with ACS, hypertension, type 2 diabetes, and renal insufficiency. He had five years of diabetes history and used oral drugs metformin and acarbose to control blood glucose. The patient was hospitalized in the Coronary Care Unit (CCU) at Renji Hospital, Shanghai on March 4, 2019 for an ACS emergency. Laboratory tests revealed the HbA1c level of 11.8%. Coronary angiography showed that this patient had diffuse lesions in the middle of the anterior descending branch with 80% stenosis, 60% stenosis of the middle segment of the circumflex artery, and 70% stenosis of the first crown of the right coronary artery. However, this patient refused to receive a percutaneous coronary intervention (PCI). At hospital admission, laboratory tests also revealed a renal insufficiency (**Table 1**). The HbA1c level of the patient was 11.8% at admission, which almost doubled the target level, and his fasting blood glucose (FBG) level ranged from 19.4 to 11.4 mmol/L from March 4 to March 6 (**Figures 1** and **2**). A clinical pharmacist was invited to participate in a consultation for blood glucose management on the afternoon of March 7. The hypoglycemic drug therapeutic program was adjusted on March 8. FBG level returned to target blood glucose range from March 9 to March 11 (**Figure 2**). Insulin therapeutic program was adjusted again on March 12 aiming for postprandial blood glucose (PBG) management after a second consultation by the clinical pharmacist the day before. With the PBG after lunch controlled within the target range and a careful assessment of the patient's physical condition, he was discharged on the afternoon of March 12. The patient did not experience hypoglycemia within one week after discharge.

**Table 1:** Results of main laboratory test and history.

Index (Normal range)	March 4^th^	5^th^	6^th^	7^th^	8 ^th^	^th^	10^th^	1^th^	12^th^
**HEMATOLOGY TESTING**									
WBC (3.69–9.16 × 10^9^/L)	14.92 ↑	−	9.90↑	10.70↑	−	−	9.00	−	−
RBC (3.68–5.12 × 10^12^/L)	4.56	−	4.26	4.67	−	−	4.71	−	−
N % (50–70%)	80.4 ↑	−	73.8 ↑	78.2 ↑	−	−	74.0 ↑	−	−
Lymphocyte % (20–40%)	5.4 ↓	−	15.2 ↓	11.3 ↓	−	−	13.8 ↓	−	−
Monocyte % (3–10%)	13.5 ↑	−	8.7	9.3	−	−	9.3	−	−
Eosinophils % (0.5–5.0%)	0.4 ↓	−	1.9	1.0	−	−	2.3	−	−
Basophil % (0.0–1.0%)	0.3	−	0.4	0.2	−	−	0.3	−	−
**SERUM LIPID INDICATORS**									
Total cholesterol (<5.72 mmol/L)	7.13 ↑	−	6.39 ↑	−	−	−	6.13 ↑	−	−
Triglycerides (<1.7 mmol/L)	11.32 ↑	−	7.44 ↑	−	−	−	3.70 ↑	−	−
LDL-C (<2.07mmol/L)	1.74	−	2.09	−	−	−	3.7 ↑	−	−
HDL-C (0.9–2.0mmol/L)	0.79	−	0.81	−	−	−	0.86	−	−
**KIDNEY FUNCTION**									
Creatinine (45–104 μmol/L)	192 ↑	−	241 ↑	242 ↑	−	−	240 ↑	−	−
Urea nitrogen (2.9–8.2 mmol/L)	12.8 ↑	−	18.5 ↑	20.2 ↑	−	−	27.7 ↑	−	−
Uric Acid (155–428 μmol/L)	578 ↑	−	690 ↑	707 ↑	−	−	658 ↑	−	−
Cystatin C (0.47–1.06 mg/L)	2.1 ↑	−	2.32 ↑	2.37 ↑	−	−	−	−	−
eGFR-EPI Cr	29 ↓	−	22 ↓	22 ↓	−	−	−	−	−
eGFR-MDRD	30 ↓	−	23 ↓	23 ↓	−	−	−	−	−
**HEPATIC FUNCTION**									
Total protein (60–83 g/L)	78.1	−	−	77.6	−	−	−	−	−
Albumin (34–54 g/L)	49.7	−	−	48.7	−	−	−	−	−
ALT (0–75 U/L)	46	−	−	24	−	−	−	−	−
AST (13–40 U/L)	165↑	−	−	23	−	−	−	−	−
T-BIL (3.4–17.1 μmol/L)	11.4	−	−	12.8	−	−	−	−	−
**MARKERS OF MYOCARDIAL INFARCTION**									
CK (30–170 U/L) CK-MB	40.9	−	−	−	−	−	−	−	−
TNI-A2 (<0.04 ng/mL)	>102 ↑	−	9.21 ↑	−	−	−	0.87 ↑	−	−
BNP (0.0–100 pg/mL)	44.4	−	−	−	−	−	−	−	−
CRP (0 ~ 3 mg/L)	<0.5	−	−	−	−	−	−	−	−

Data were collected from March 4^th^ 2019 to March 12^th^ 2019. −, Not Tested; WBC, White Blood Cell; RBC, Red Blood Cell; N%, Neutrophile Granulocyte %; eGFR, estimated glomerular filtration rate; DL-C, Low-Density Lipoprotein Cholesterol; HDL-C, High-Density Lipoprotein Cholesterol; ALT, Alanine Amino Transferase; AST, Aspartate Amino Transferase; T-BIL, Total Bilirubin; CK, Creatine Kinase; TNI-A2, Troponin; BNP, Brain Natriuretic Peptide; CRP, C-Reactive Protein. ↑ means above the normal upper limit and ↓ means below the normal lower limit.

## Investigations

Clinical investigations of the patient's vital signs, coadministrated drugs, and blood glucose levels were performed to assess the treatments for the patient.

### Laboratory Tests

Laboratory tests were performed in the department of clinical laboratory. Test results of hematology parameters, serum lipid indicators, kidney function, hepatic function, and myocardial infarction markers were listed in **Table 1**. The patient had normal hepatic functions but was diagnosed with renal insufficiency as indicated by an estimated glomerular filtration rate (eGFR) less than 30 ml/min/1.73m^2^ (Chronic kidney disease stage 4) (Levey et al., 2003; Webster et al., 2017). In addition, the patient had hyperuricemia, although did not develop uric acid-related symptoms. Notably, his blood troponin level was reduced from above 102 on day one of hospitalization to 0.87 ng/ml on day seven, suggesting an effective cardiac rescue (**Table 1**).

### Drugs Therapeutic Program

The main drugs' therapeutic program for this patient during CCU hospitalization was listed in **Table 2**. Since the patient suffered from ACS but refused to receive PCI, Oral dual antiplatelet therapy (DAPT) and hypolipidemic therapy were administered as conservative therapy. Good management of the patient's blood glucose could effectively reduce the occurrence of cardiovascular events. To lower the blood glucose level in this patient, both oral and injectable hypoglycemic drugs were used. For hospitalized patients, insulin is often more valuable than oral hypoglycemic drugs when the patients have indications for insulin usage. In this patient, Novolin N was used to lower FBG, and regular insulin injection was used to control PBG. When PBG was still higher than expected, regular insulin injection was replaced by temporary insulin injection.

**Table 2:** Main medications information and history.

Medications	March 4^th^	5^th^	6^th^	7^th^	8^th^	9^th^	10^th^	11^th^	12^th^
**HYPOGLYCEMIC DRUGS**									
**Oral hypoglycemic drugs**									
Metformin	0.5 g *tid*								
Acarbose	50 mg *tid*								
Canagliflozin			100 mg *qd*						
**Injected hypoglycemic drugs**									
Insulin^‡^		8 U–8 U–8 U			5U–5 U–5 U				8 U–8 U–5 U
Novolin N		6 U			14 U				
Total temporary insulin	24 U	12 U	8 U	6 U	6 U	10 U			
**ORAL HYPOLIPIDEMIC DRUGS**									
Atorvastatin	20 mg *qn*								
Pravastatin		10 mg *qn*							
Fenofibrate		200 mg *qd*							
**ORAL ANTIPLATELET DRUGS**									
Aspirin	100 mg *qd*								
Clopidogrel	75 mg *qd*								
**ORAL DIURETICS**									
Furosemide	20 mg *qd*								
Spirolactone	20 mg *qd*								
**ORAL PROTON PUMP INHIBITOR**									
Pantoprazole	40 mg *qd*								
**ORAL ANTIHYPERTENSIVE DRUGS**									
Metoprolol	12.5 mg *qd*								
Benazepril	2.5 mg *qd*								

^‡^Routine insulin used 30 min ahead of breakfast, lunch, and supper. Data were collected from March 4^th^ 2019 to March 12^th^ 2019. Tid, ter in die; qd, quaque die; qn, quaque nocte. Colored shadings means the specific use time of various drug.

### Blood Glucose Levels

**Figure 3:**
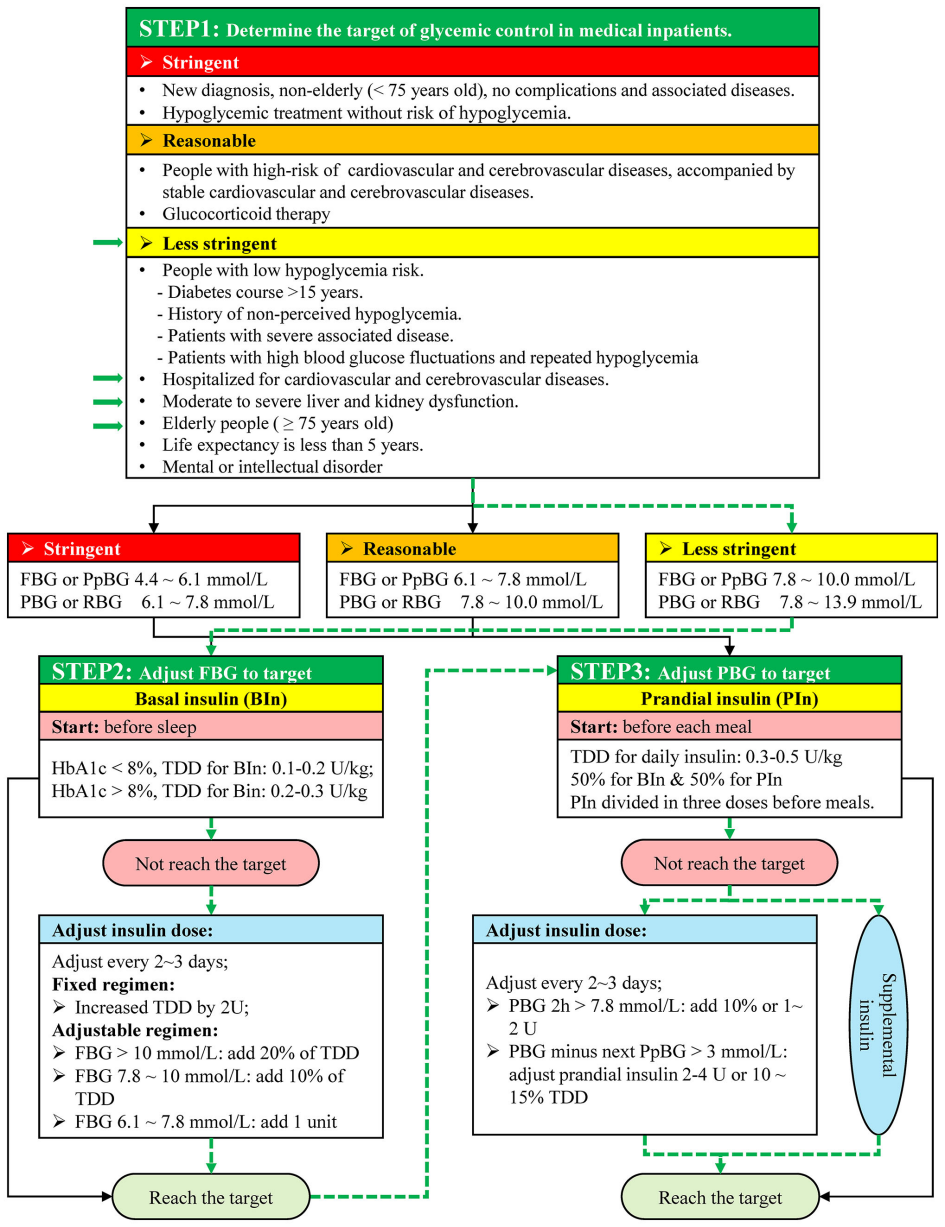
Blood glucose adjustment strategy. FBG, Fasting Blood Glucose; PpBG, Preprandial Blood Glucose; PBG, Postprandial Blood Glucose; RBG, Random Blood Glucose; TDD, Total daily dose; HbA1c, glycosylated hemoglobin; BIn, Basal insulin; PIn, Prandial insulin. The green arrows indicate patient information and blood glucose adjustment in this case.

Blood glucose levels were detected by using Contour TS blood glucose meter (Bayer HealthCare) and Contour TS blood glucose test strips. Blood glucose levels were tested by four times a day (6:00 for FBG, 9:00 for breakfast PBG, 13:00 for lunch PGB, and 19:00 for dinner PBG). Dynamic changes of blood glucose and insulin dosage used were shown in **Figures 1** and **2**. This patient was hospitalized for cardiovascular disease, so the target of glycemic control of this patient should be less stringent (**Figure 3**). The patient started with an FBG level of 19.4 mmol/L but achieve the less stringent FBG target range (7.8–10 mmol/L) within two days after the first therapeutic program adjustment by increasing the dose of Novolin N from 6 U to 14 U while decreasing the dose of insulin from 8 U to 5 U. The level of PBG 2 h after lunch, which was presented as the highest blood glucose parameter of the patient on day 1 (28.2 mmol/L), was successfully reduced to the less stringent PBG target range (7.8–10 mmol/L) on the same day as the second therapeutic program adjustment (**Figure 2**). Importantly, temporary insulin was not used in the second half of the treatment phase but the blood glucose was well controlled in this patient.

## Discussion

### Diabetes, Cardiovascular Disease, and Chronic Kidney Disease

T2D has contributed tremendously to the mortality and disability worldwide (Zheng et al., 2018). It was estimated that there are more than 415 million adults worldwide and 109.6 million adults in China who are living with diabetes (Zheng et al., 2018). Cardiovascular disease and renal disease are two major complications of T2D. About half of the T2D patients have microvascular complications, and about a quarter of patients with T2D have developed macrovascular complications (Litwak et al., 2013). Hyperglycemia induced superoxide overproduction by the mitochondrial electron transport chain *via* polyol pathway flux, advanced glycation end products, protein kinase C, and the hexosamine pathway is the main reason that T2D is implicated in vascular damage and cardiac myocyte death (Sasso et al., 2018). Several studies suggested that diabetes could be an independent predictor of new cardiovascular events (Sasso et al., 2018; Avogaro et al., 2019). In-hospital appropriate glucose control has a neutral effect on mortality after ACS (Avogaro et al., 2019). Compared with patients in developed countries, patients with T2D in most developing countries have a higher risk of developing kidney complications (Zimmet et al., 2014). Overall, cardiovascular disease, diabetes, and chronic kidney disease (CKD) are the main cause leading to death in Asia (Misra et al., 2017). The definition and classification of CKD have evolved over time (Levey et al., 2003; Webster et al., 2017). CKD is currently defined as GFR less than 60 ml/min/1.73m^2^ for at least 3 months duration (Webster et al., 2017). Patients with CKD have 57% higher cardiovascular mortality and 33% higher risk of developing a nonfatal myocardial infarction (Webster et al., 2017). The diabetes patient in the present study was accompanied with severely decreased eGFR, categorizes as chronic kidney disease stage 4 (GFR ranged from 15 to 29 ml/min/1.73m^2^), therefore had extremely high risk of cardiovascular mortality (Webster et al., 2017). In addition, this patient should be considered as an intensive care subject due to the five-year T2D history and present ACS. Besides antiplatelet therapy, effective control of blood glucose was pivotal for this patient during the hospitalization.

### Management of Blood Glucose in Hospitalized Patients

The eGFR of the patient in this study was 23 ml/min/1.73m^2^. For a patient with renal insufficiency, metformin and acarbose are contraindicated if eGFR is less than 30 ml/min/1.73m^2^, and sodium-glucose cotransporter 2 inhibitors (SGLT2i) such as canagliflozin are not suitable when eGFR is less than 45 ml/min/1.73m^2^ (Association and Association, 2016; Garber et al., 2019). Based on the Standards of Medical Care in Diabetes-2019, when blood glucose levels are higher than 16.7 mmol/L (≥300 mg/dl), the early introduction of insulin should be considered (Association, 2019c). Based on Chinese endocrinologist consensus on blood glucose management for Chinese inpatients, different blood glucose control targets should be set for hospitalized patients under different health conditions (Chinese Endocrinologist and inpatients, 2017). According to a patient's condition and the reason for hospitalization, the glycemic goals are divided into three levels, namely the stringent level, reasonable level, and less stringent level (**Figure 3**) (Chinese Endocrinologist and inpatients, 2017; Association, 2019b; Garber et al., 2019; Umpierrez et al., 2012). The first step of blood glucose management is to determine the glycemic target of hospitalized patient. This patient was a 75-year old male who was hospitalized for cardiovascular disease accompanied with kidney dysfunction. The glycemic target for this patient was therefore less stringent based on international standards of medical care in diabetes and Chinese expert consensus (Chinese Endocrinologist and inpatients, 2017; Association, 2019b). With the glycemic target level set, the second step was to adjust FBG to its target. The target range of FBG or preprandial blood glucose (PpBG) is 7.8 to 10.0 mmol/L. By using basal insulin (BIn) before sleep, the FBG level target was gradually achieved. The starting total daily dose (TDD) of BIn was suggested with a range of 0.2 to 0.3 U/kg in patients with HbA1c above 8% (Umpierrez et al., 2012; Garber et al., 2019). BIn dose should be adjusted every 2 to 3 days if the FBG does not reach the target (**Figure 3**) (Garber et al., 2019). In this patient, the FBG level target was achieved on the second day after the first therapeutic program adjustment. The improvements in ACS and subsequent decreases in sympathetic nervous system activation and cortisol induced gluconeogenesis also contributed to the reduction in FBG (Angeli et al., 2015). The third step after the FBG level has reached the target aims to adjust the PBG to its target. Nutritional management is also very important in the management of T2D (Russell et al., 2016). Russell et al. published a review of the impact of diet composition on blood glucose regulation in 2016, which is still very valid (Russell et al., 2016). Dietary carbohydrates, fiber, protein, vitamins, minerals, gut microbiota, and other compositions could affect blood glucose levels (Russell et al., 2016). A low Glycemic Index diet can improve glycemic control, which could reduce interleukin 6 expression caused by postprandial glycemic fluctuations (Russell et al., 2016). The diet of this patient during hospitalization was diabetes diet (Sato et al., 2004). The diet can improve blood glucose to a certain extent, but insulin use during hospitalization is essential for T2D patients. Half of TDD of insulin should be used as prandial insulin (PIn) and divided in three doses before meals (Ruiz de Adana et al., 2015; Garber et al., 2019). In this patient, the glycemic goal of PBG was 7.8 to 13.9 mmol/L. In order to target the FBG goal, PIn before three meals was a simple and easy operating method. On the second day when this patient's FBG target was achieved, a clinical pharmacist was invited for the second consultation, and treatment adjustment was performed on the following day. Insulin dose was changed from 5U to 8 U and was used before breakfast and lunch. In order to prevent fasting hypoglycemia, the insulin dose before dinner did not change. The patient's PBG reached the target after his lunch on the same day of adjustment. Given the well-controlled blood glucose level and stabilized cardiac condition, the patient was then discharged. Before this patient left the hospital, the clinical pharmacist carried out diabetes education, which included diet education, sports education, and medication education, to help him improve the quality of life back home. The patient did not have hypoglycemia during the following week after discharge, and the blood glucose control was effective. Hence, we believe this glycemic management protocol (**Figure 3**) could be used to quickly adjust blood glucose in hospitalized patients, especially in patients with cardiovascular complications.

### Model of Management Coordinated by Clinical Pharmacists and Clinicians

Chinese clinical pharmacists are playing an increasingly important role in clinical treatment and patients' management. After one-year formal training at the National Clinical Pharmacist Training Bases, more and more specialist clinical pharmacists are involved in the clinical treatment process. Specialist clinical pharmacists work with specific clinical departments and participate in the formulation and adjustment of medication treatment programs. In addition, specialist clinical pharmacists also participate in the consultation of drug-related treatment strategies in other clinical departments of the hospital. Besides clinical consultation, medication education programs for patients also form a core management strategy for clinical pharmacists. In this case report, we not only report a simple glycemic management protocol for hospitalized diabetes patients, but also provide a new model of cooperation between clinical pharmacists and clinicians.

Our case report had a few potential limitations. Firstly, the diet composition can directly affect the blood glucose level, but we did not make a specific analysis of the patient's dietary composition during hospitalization. Secondly, improvements in ACS and subsequently reduced stress level could also contribute to the overall decrease in FBG through the sympathetic nervous system and the hypothalamic–pituitary axis (Angeli, 2015), but the lack of effective controls in our case report limited our capacity to investigate the potential effects from it.

## Concluding Remarks

The glycemic management strategy in this case report is based on guidelines for blood glucose management in China and the United States. We recommend this management model for the blood glucose control in hospitalized patients, particularly those with cardiovascular complications. In addition, the contribution of clinical pharmacists in the clinical treatment process has become increasingly prominent. The treatment model cooperated by clinical pharmacists and clinicians is the trend of future development. Experiences based on a single patient are limited for instant generalizations, but this study possibly provides a good starting point.

## Data Availability Statement

The raw data supporting the conclusions of this article will be made available by the authors, without undue reservation, to any qualified researcher.

## Ethics Statement

The studies involving human participants were reviewed and approved by Shanghai Jiaotong University School of Medicine, Renji Hospital Ethics Committee (KY-2019-076). The patients/participants provided their written informed consent to participate in this study.

## Author Contributions

Conceptualization: F-HS designed the blood glucose adjustment strategy. LS was in charge of the treatment of this patient. F-HS and M-MP were responsible for collecting the patient's information. F-HS and CZ were involved in the care of the patient and interpreted the results. L-LM polished the language. HL wrote the manuscript, designed and drew the figures. Z-CG and JM supervised the investigation, interpreted the results, and revised the manuscript.

## Funding

This article is supported by “the Clinical Pharmacy Innovation Research Institute of Shanghai Jiao Tong University School of Medicine (2019)” (CXYJY2019QN004 and CXYJY2019ZD001), “the Fundamental Research Funds for the Central Universities” (17JCYB11), “the Pharmaceutical Fund of College Of Medicine, Shanghai Jiao Tong University” (JDYX2017QN003), “Program for Key Discipline of Clinical Pharmacy of Shanghai” (2016-40044-002), “Program for Key but Weak Discipline of Shanghai Municipal Commission of Health and Family Planning” (2016ZB0304), the Scientific Research Program of China Hospital Development Research Institute, Shanghai Jiao Tong University (No. CHDI-2019-A-01 and No. CHDI-2019-B-17).

## Conflict of Interest

The authors declare that the research was conducted in the absence of any commercial or financial relationships that could be construed as a potential conflict of interest.

## Abbreviations

ACS, acute coronary syndromes; CCU, Coronary Care Unit; FBG, fasting blood glucose; PBG, postprandial blood glucose; T2D, type 2 diabetes; RBG, random blood glucose; PpBG, preprandial blood glucose; TDD, total daily dose; HbA1c, hemoglobin A1c; Bin, basal insulin; Pin, prandial insulin; eGFR, estimated glomerular filtration rate; T2D, type 2 diabetes; PCI, percutaneous coronary intervention; DAPT, dual antiplatelet therapy; CKD, chronic kidney disease; SGLT2i, sodium-glucose cotransporter 2 inhibitors.
